# Supplementary material for: Shading Treatment Reduces Grape Sugar Content by Suppressing Photosynthesis-Antenna Protein Pathway Gene Expression in Grape Berries
Source: Int J Mol Sci. 2024 May 5;25(9):5029. doi: 10.3390/ijms25095029 (PMC11084848; doi:10.3390/ijms25095029)
Supplement: Supplementary file 1 [file ijms-25-05029-s001.zip › ijms-2949822-supplementary.pdf]

Table S1 Eight differential genes were used for primer sequence of qRT-PCR.

Gene name		Primer sequence
VIT-12s0055g01110 (Lhcb6)	VIT_12s0055g01110-F	TGGGCTCTCCGTTCTTGACTG
	VIT_12s0055g01110-R	GGCAGCAGCGACGACAATG
VIT_08s0007g02190 (Lhcb4)	VIT_08s0007g02190-F	CCGCCACTCCTTACACAACAG
	VIT_08s0007g02190-R	TTTCTTACGACCGAACCCGAATC
VIT_19s0014g03660 (Lhcb3)	VIT_19s0014g03660-F	CCGCTGGATTGTCTGCTGATC
	VIT_19s0014g03660-R	CTAAGCCACTTCTCAAGGACTTCTG
VIT_12s0057g00630 (Lhcb2)	VIT_12s0057g00630-F	GCAGAACTGAAGGTGAAGGAGATC
	VIT_12s0057g00630-R	TGACAATAGCCTGAACGAAGAATCC
VIT_10s0003g02900 (Lhcb1)	VIT_10s0003g02900-F	GCAGAGCCTTACTTACTTGGATGG
	VIT_10s0003g02900-R	ACCGCCCGTTGATGACCTC
VIT_15s0024g00040 (Lhca3)	VIT_15s0024g00040-F	CCACCATCTCTCCCAGTTCTCAG
	VIT_15s0024g00040-R	ACTTCTTCAGGCTCAGTCTCTTCC
VIT_01s0010g03620 (Lhca2)	VIT_01s0010g03620-F	CCACCATCTCTCCCAGTTCTCAG
	VIT_01s0010g03620-R	ACTTCTTCAGGCTCAGTCTCTTCC
VIT_13s0019g04140 (Lhca1)	VIT_13s0019g04140-F	ATGGAGAAGGACACCGAGAAGAAG
	VIT_13s0019g04140-R	ACGCAGAACCCGACGAACG
Primer VvGAPDH	VvGAPDH-F	TTCTCGTTGAGGGCTATTCCA
parameters	VvGAPDH-R:	CCACAGACTTCATCGGTGACA
